# Investigation on the Gradient Nanomechanical Behavior of Dental Fluorosis Enamel

**DOI:** 10.1186/s11671-018-2768-y

**Published:** 2018-10-30

**Authors:** Jie Min, Ping Yu, Zhou Xu, Zhi Li, Qianqian Zhang, Haiyang Yu, Shanshan Gao

**Affiliations:** State Key Laboratory of Oral Diseases, National Clinical Research Center for Oral Diseases, West China Hospital of Stomatology, Sichuan University, Chengdu, China

**Keywords:** Dental fluorosis, Enamel, Microstructure, Nanoindentation, Nanoscratch

## Abstract

This study aims to investigate the gradient nanomechanical behavior of dental fluorosis enamel and provide appropriate selection criteria for restorative materials. The nanomechanical properties of the outer, middle, and inner layers of normal tooth enamel, mild dental fluorosis enamel, and severe dental fluorosis enamel were tested by nanoindentation under an applied load of 2000 μN and holding time of 30 s. The nanotribological properties were then evaluated through nanoscratch tests under an applied load of 1000 μN. In addition, the nanotribological property of the outer layer of dental fluorosis enamel was compared with that of four restorative materials, namely, lithium disilicate glass-ceramic (IPS e.max CAD), polymer-infiltrated-ceramic network (PICN), composite resin block (Lava™ ultimate), and conventional composite resin (Fltek™ Z350XT). The nanohardness and elastic modulus of mild dental fluorosis enamel increased from the outer to the middle layers and then decreased from the middle to the inner layers. By contrast, the changed displacement, friction coefficient, and nanoscratch depth and width decreased from the outer to the middle layers and then increased from the middle to the inner layers. In severe dental fluorosis enamel, nanohardness and elastic modulus increased from the outer to the inner layers, but the changed displacement, friction coefficient, and nanoscratch depth and width decreased from the outer to the inner layers. The nanoscratch depth and width of Lava™ ultimate were similar to those of the outer layer of the mild dental fluorosis enamel. The gradient nanomechanical behavior of dental fluorosis enamel significantly differed from that of normal tooth enamel. Dental materials with a wear resistance similar to that of the opposing enamel are a good choice for restoring dental fluorosis (trial registration: WCHSIRB-D-2014-126, registered 25 December 2014).

## Introduction

**Fig. 1:**
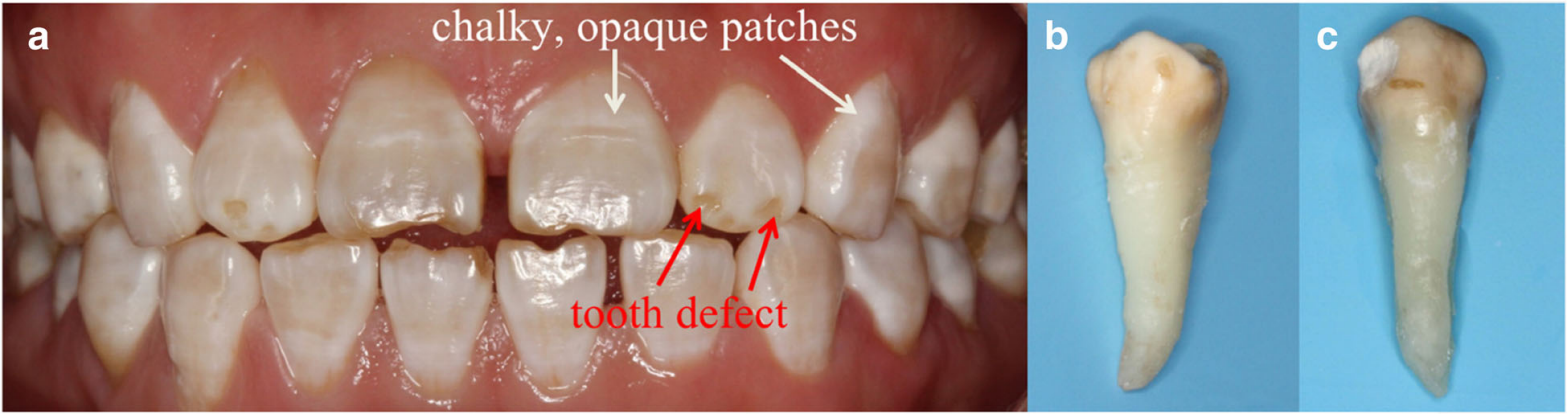
Photographs of dental fluorosis. **a** Intraoral photograph of mild dental fluorosis showing chalky, opaque patches and severe dental fluorosis showing both chalky, opaque patches and tooth defects. **b** Extracted mild dental fluorosis. **c** Extracted severe dental fluorosis

Dental fluorosis is a tooth malformation caused by the intake of excess fluoride from various sources, such as water, food, and air, during tooth development and mineralization [1, 2]. The regional concentration of fluoride and extensive application of fluoride to prevent dental caries have resulted in the high incidence of this malformation. The incidence of dental fluorosis reaches 80–90% in some high-fluoride areas [3, 4]. Dental fluorosis is characterized by the presence of chalky, opaque patches or tooth defects that affect the appearance and function of the teeth (Fig. 1a). This condition may further result in serious mental burden and socialization barrier [5]. Patients with dental fluorosis often require restoration to recover their dental appearance and function [6, 7]. Matching of the mechanical and tribological properties of the dental restoration to those of the opposing tooth enamel is very important to achieve good clinical outcomes [8, 9]. Mismatches between material properties can cause the excessive wear of the opposing natural tooth or the restoration itself [10, 11]. Thus, a thorough investigation of the microstructure, nanomechanical properties, and nanotribological properties of dental fluorosis enamel is necessary to select the appropriate restorative materials [12].

The outermost layer of the enamel protects the dentin and vital pulp from the oral environment. The dental enamel should be able to withstand the mastication forces over millions of cycles throughout the entire lifetime of an individual [13–15]. It must exhibit superior mechanical properties to dissipate stress in the tooth and to prevent crack initiation [12]. Given that the microstructure and composition of enamel change from the outer enamel toward the enamel-dentin junction (EDJ), the natural tooth enamel exhibits gradient mechanical behaviors [15–18]. Chronic exposure to high fluoride levels results in structural changes in dental enamel and leads to dental fluorosis [19–21]. These changes are often accompanied by alterations in the mechanical behavior of the enamel [22–24]. Shearer et al. [22] and Suckling et al. [23] used an animal model to study the mechanical behavior of the dental fluorosis enamel. Fan et al. [24] investigated the mechanical behavior of human mild dental fluorosis enamel. To date, however, the gradient nanomechanical behavior of the dental fluorosis enamel remains unclear. In addition, the selection criteria for restorative materials for dental fluorosis are also ambiguous. Therefore, this study investigates the gradient nanomechanical behavior of the mild dental fluorosis enamel and severe dental fluorosis enamel. The nanotribological properties of four different restorative materials are compared with those of the outer layer of the dental fluorosis enamel. The results of this study will guide the clinical selection and development of restorative materials for dental fluorosis.

## Materials and Methods

A total of 30 caries-free premolars (10 normal teeth, 10 mild dental fluorosis teeth showing chalky, opaque patches [Fig. 1b] and 10 severe dental fluorosis teeth showing chalky, opaque patches and tooth defects [Fig. 1c]) were collected. The ages of the donors ranged from 19 to 25 years. All donors with dental fluorosis had lived in areas with high fluorine concentration. The study protocol was approved by the Ethics Committee of West China Hospital. After extraction, the teeth were stored in Hank’s balanced salt solution (HBSS, Solarbio, Beijing, China) at 4 °C to prevent dehydration and demineralization prior to sample preparation. All samples were tested within 1 week after extraction.

### Sample Preparation

Tooth crowns were separated from the roots by using a high-speed cutting machine (Struers Minitom, Struers, Denmark) with a diamond abrasive cutoff wheel (Struers, Denmark) operating at 300 rpm under water irrigation. The crowns were then cut into two halves and embedded in epoxy resin (EpoFix, Struers, Denmark) with their longitudinal sections exposed. One half of the crown was used for the nanoindentation tests, and the other half was used for the nanoscratch tests. Five specimens (4 mm × 4 mm × 2 mm) for each restorative material [lithium disilicate glass-ceramic (IPS e.max CAD) (Ivoclar Vivadent AG), polymer-infiltrated-ceramic networks (PICN) (Vita Zahnfabrik, Bad Sackingen, Germany), composite resin block (Lava™ ultimate) (3M ESPE, Seefeld, Germany), and conventional composite resin (Fltek™ Z350XT) (3M ESPE, MN, USA)] were also prepared. The specimens were sequentially polished, beginning with #800 mesh SiC paper (silicon carbide paper, Struers) and then with increasingly finer abrasives until #4000 mesh. Thereafter, the specimens were polished with 3 μm and 0.04 μm abrasive particle solutions (OP-S NonDry, Struers, Denmark) with a water base. Finally, the specimens were ultrasonically cleaned for 15 s. In this study, the enamel was divided into three layers, namely, the outer enamel, which has a maximum distance of at most 100 μm from the occlusal surface; the middle enamel, which is located midway between the occlusal surface and the EDJ (middle enamel); and the inner enamel, which has a maximum distance of at most 100 μm from the EDJ (inner enamel) [25].

### Nanoindentation Tests

Nanoindentation tests were performed by using a nanoindentation device (Triboindenter TI950, Hysitron, USA) with a Berkovich diamond indenter (nominal radius of ~ 150 nm). In situ scanning probe microscope (SPM) was equipped in the nanoindentation system in order to accurately locate different areas of the tooth enamel. The indents were performed under an applied load of 2000 μN and holding time of 30 s. The rate of loading and unloading was 400 μN/s. Fifty indents were performed on each enamel layer of the normal tooth, mild dental fluorosis, and severe dental fluorosis. The distance between indents was set to over 5 μm. The reduced elastic moduli and nanohardness were measured through the conventional Oliver and Pharr approach [26, 27]. The contact displacements before and after the holding time were recorded. Then, the changed displacement was calculated by subtracting the initial depth at the beginning of the holding time from the penetration depth at the end of the holding period under the maximum load. The changed displacement was used to assess the nanoindentation creep response.

### Nanoscratch Tests

Nanoscratch tests were performed using a nanoscratch device (Triboindenter TI950, Hysitron, USA), with a conical diamond indenter (nominal radius of ~ 1 μm) (Hysitron Triboscope, MN, USA). The scratches were applied under a load of 1000 μN at a rate of 0.5 μm/s and scratch length of 10 μm. Fifty scratches were applied in each enamel layer of normal tooth enamel, mild dental fluorosis enamel, and severe dental fluorosis enamel, as well as the restorative materials. The distance between scratches was set to over 5 μm. After the nanoscratch tests, the friction coefficient and nanoscratch depth and width were recorded by the system.

### Statistical Analysis

Statistical analyses were performed using SPSS 18.0. One-way ANOVA and Student’s *t* tests were performed to analyze the data. A *p* value of less than 0.05 was considered statistically significant.

### SEM Observation

The microstructures of the three enamel layers of the normal tooth, mild dental fluorosis, and severe dental fluorosis were investigated by field emission gun scanning electron microscopy (SEM) (INSPECT F, Czech Republic).

## Results and Discussion

### Microstructure and Gradient Nanomechanical Behavior of Dental Fluorosis Enamel

**Fig. 2:**
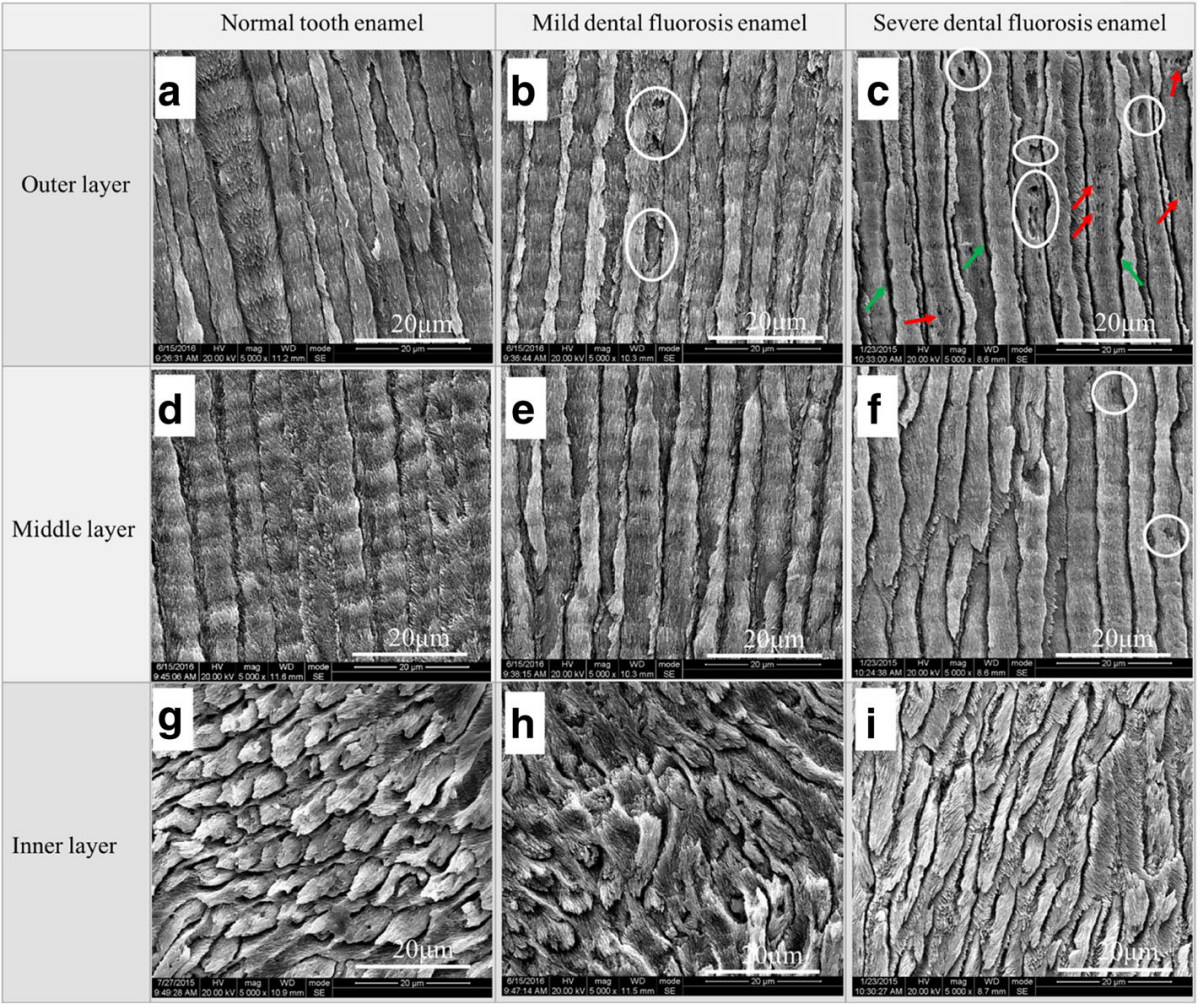
SEM images of the normal tooth enamel, mild dental fluorosis enamel, and severe dental fluorosis enamel. The **a**–**c** outer layers, **d**–**f** middle layers, and **g**–**i** inner layers were etched with 37% phosphoric acid for 30 s and then visualized under × 5000 magnification. Green arrows indicate broadened gaps between the enamel rods, while white circles indicate pores. Red arrows indicate loosely arranged crystals in the enamel rods with increasing crystal clearance and micropores

The microstructures of the three enamel layers of the normal tooth, mild dental fluorosis, and severe dental fluorosis are shown in Fig. 2. The outer and middle enamel rods of the normal teeth exhibited uniform diameters and were arranged in an upright manner (Fig. 2a, d), while their inner enamel rods presented an undulating or weaving pattern (Fig. 2g). In mild dental fluorosis, a small number of pores (white circles in Fig. 2b) were observed on the outer enamel, but their middle and inner layers (Fig. 2e, h) presented microstructures similar to those of the normal teeth. The structure of the outer enamel rods of severe dental fluorosis was characterized by broadened gaps between the enamel rods (green arrow in Fig. 2c) and numerous pores (white circles in Fig. 2c). Crystals in the enamel rod were loosely arranged with increasing crystal clearance and micropores (red arrow in Fig. 2c). A small number of pores (white circles in Fig. 2f) were also found in the middle layer. The structure of the inner enamel of severe dental fluorosis was similar to that of the normal teeth (Fig. 2i). Compared with those of the normal teeth, the microstructures of the outer enamel of mild dental fluorosis and the outer and middle enamel of severe dental fluorosis showed marked differences, which could be attributed to two factors [28–31]. One factor is the interference of excessive fluoride intake during the normal formation of tooth enamel at puberty. This process results in excessive matrix protein retention, enamel rod hypomineralization, and a loose crystalline arrangement of the enamel rods [28–30]. The other factor is the chemical change in hydroxyapatite crystals caused by excessive fluoride intake. Fluoride apatite is formed when fluoride element displaces the hydroxyl in hydroxyapatite crystals [31].

**Fig. 3:**
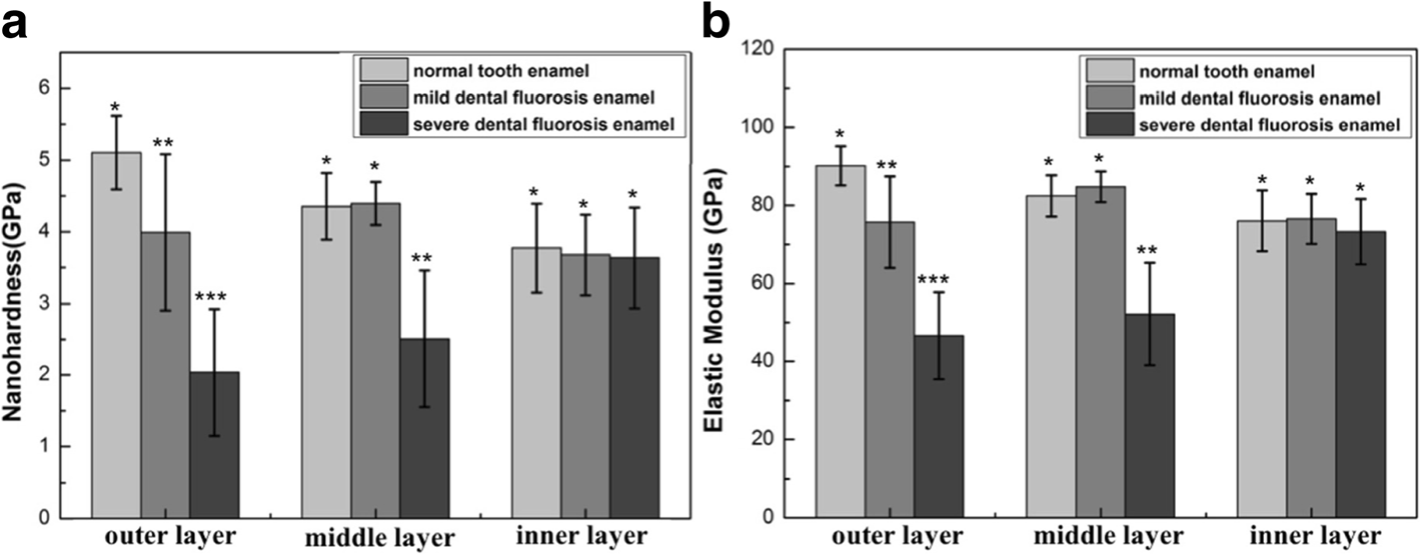
Nanomechanical properties of the enamel layers of the normal tooth, mild dental fluorosis, and severe dental fluorosis. **a** Nanohardness. **b** Elastic modulus. Identical symbols denote no significant difference in nanohardnesses and elastic moduli among the corresponding layers of the normal tooth enamel, mild dental fluorosis enamel, and severe dental fluorosis enamel

**Fig. 4:**
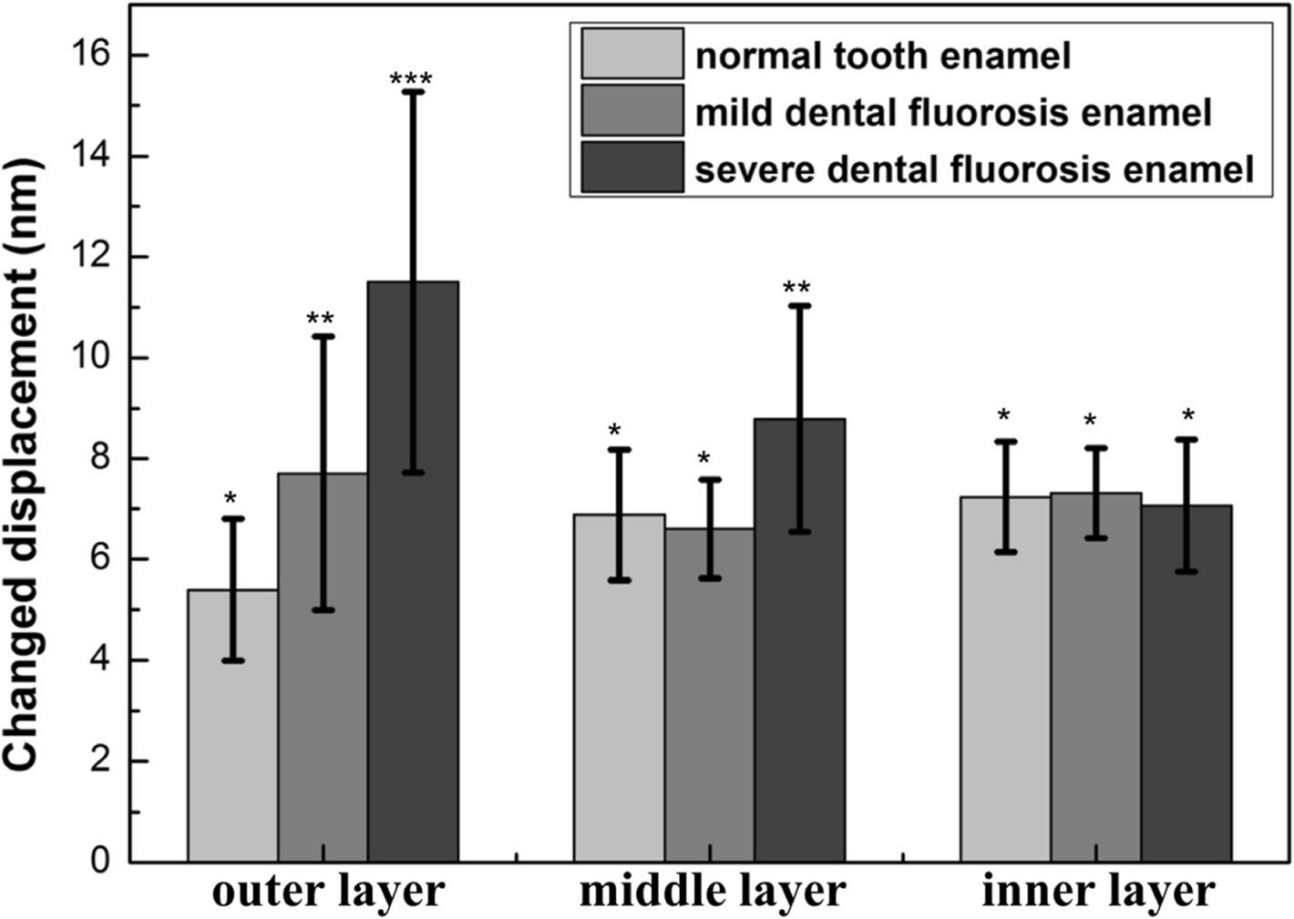
Nanoindentation creep behavior of the enamel layers of the normal tooth, mild dental fluorosis, and severe dental fluorosis. Identical symbols denote no significant difference in nanoindentation creep behavior among the corresponding layers of the normal tooth enamel, mild dental fluorosis enamel, and severe dental fluorosis enamel

In the normal tooth enamel, the nanohardness and elastic modulus decreased from the outer to the inner layers (Fig. 3), whereas the changed displacement increased from the outer to the inner layers (Fig. 4). The orientation of the enamel rods and chemical components resulted in the gradient nanomechanical properties from the outer to the inner layers of the tooth enamel [32–34]. Normal tooth enamel presented a complex hierarchical structure [18, 35]. The outer enamel rods were straight and aligned parallel to one another, while the inner enamel rods extended within alternating “bands” [36]. During mastication, stress extends along the upright rods (outer enamel) until the available energy is drained or deflected by the decussated enamel (inner enamel) [36]. The tooth enamel consists of 96% mineral materials, 1% organic protein, and 3% water by weight, and organic protein contents increase from the outer enamel to the EDJ [37]. The organic components of the teeth promote antifatigue responses and contribute to crack arrest [38, 39], and the formation of ligament bridges of organic protein also promotes closure stresses [40]. Due to the differences in their microstructures (Fig. 2) and increased organic content [41], dental fluorosis enamel showed gradient nanomechanical behaviors different from those of the normal tooth enamel. The nanohardness and elastic modulus of the mild dental fluorosis enamel increased from the outer to the middle layers and then decreased from the middle to the inner layers (Fig. 3). The changed displacement (7.70 ± 2.71 nm) of the outer layer of the mild dental fluorosis enamel was significantly larger than that of the normal tooth enamel (*p* < 0.05), and the changed displacement decreased from the outer to the middle layers and then slightly increased from the middle to the inner layers (Fig. 4). For the severe dental fluorosis enamel, the nanohardness and elastic modulus increased from the outer to the inner layers. The nanohardness (2.04 ± 0.89 GPa) and elastic modulus (46.63 ± 11.19 GPa) of the outer layer of the severe dental fluorosis enamel were lower than those of its middle layer, and the inner layer showed the highest values among those layers (*p* < 0.05) (Fig. 3). The changed displacement of severe dental fluorosis enamel decreased from the outer to the inner layers, and the changed displacement (11.50 ± 3.77 nm) of the outer layer was larger than that of the middle layer (8.79 ± 2.24 nm). Among the layers, the inner layer showed the lowest displacement (*p* < 0.05) (Fig. 4).

**Fig. 5:**
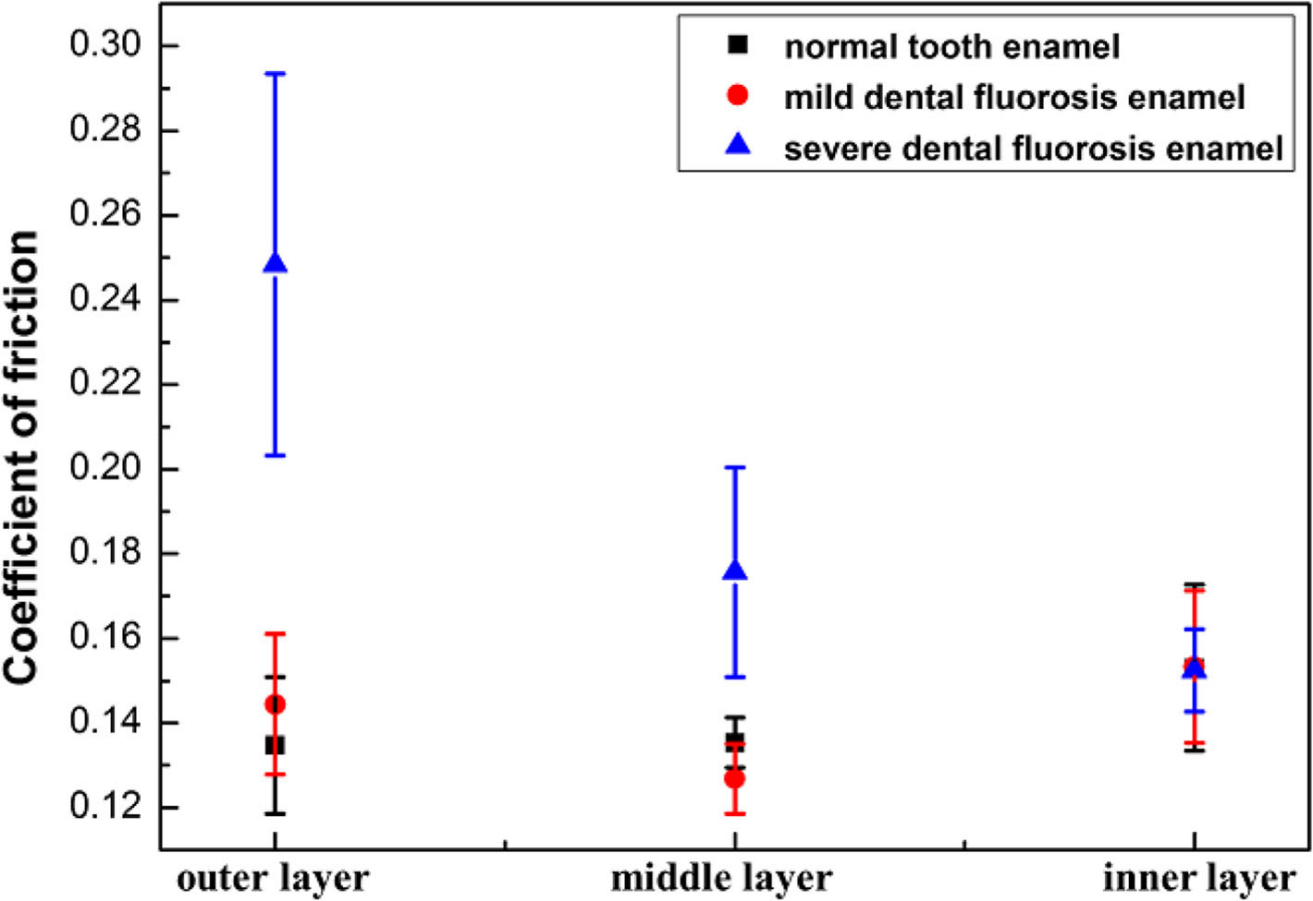
Friction coefficients of the enamel layers of the normal tooth, mild dental fluorosis, and severe dental fluorosis

The friction coefficients of the three enamel layers of the normal tooth, mild dental fluorosis, and severe dental fluorosis are shown in Fig. 5. The friction coefficient of the normal tooth enamel increased from the outer to the inner layers. In mild dental fluorosis enamel, the friction coefficient decreased from the outer to the middle layers and then increased from the middle to the inner layers. In severe dental fluorosis enamel, the friction coefficients of the outer (0.25 ± 0.044) and middle (0.18 ± 0.025) layers were significantly larger than those of the mild dental fluorosis enamel and normal tooth enamel (*p* < 0.05). In addition, the friction coefficient of the severe dental fluorosis enamel decreased from the outer to the inner layers (*p* < 0.05).

**Fig. 6:**
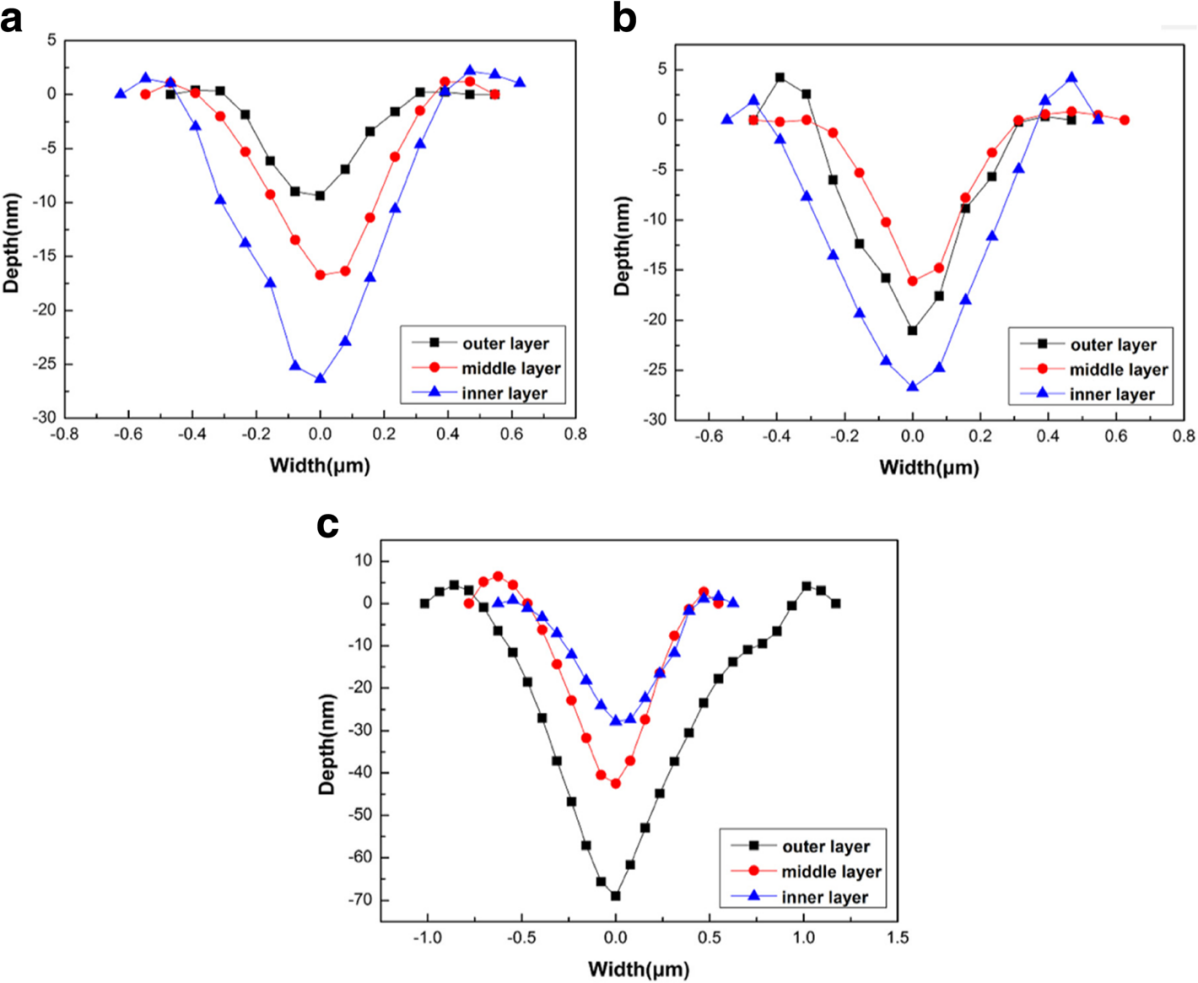
Profiles of nanoscratch tracks on the enamel layers of the normal tooth, mild dental fluorosis, and severe dental fluorosis. **a** Normal tooth enamel. **b** Mild dental fluorosis enamel. **c** Severe dental fluorosis enamel

The nanoscratch depths and widths of the three enamel layers of the normal tooth, mild dental fluorosis, and severe dental fluorosis are shown in Fig. 6. Normal tooth enamel showed a nanoscratch depth and width that increased from the outer to the inner layers (Fig. 6a), While the mild dental fluorosis enamel revealed a nanoscratch depth and width that decreased from the outer to the middle layers and then increased from the middle to the inner layers (Fig. 6b). Variations in the nanoscratch depth and width of the severe dental fluorosis enamel were significantly different from those of the normal tooth enamel. Specifically, nanoscratch depths and widths decreased from the outer to the inner layers of severe dental fluorosis enamel (Fig. 6c).

The wear resistance of the normal tooth enamel decreased from the outer to the inner layers, and this behavior corresponds with that observed in previous studies [42–44]. Excess fluoride can form fluoride-like deposits on the enamel surface and reduce wear resistance [3, 45, 46]. In this study, the wear resistances of the outer and middle layers of severe dental fluorosis enamel and the outer layer of mild dental fluorosis enamel were remarkably lower than those of the normal tooth enamel. The inter-rod enamel contains more protein than the enamel rod, acts as a buffer layer that absorbs and disperses the pressure on the tooth, and affects the wear resistance of the tooth enamel [43]. Excessive fluoride intake leads to hypomineralized enamel rod formation and excessive matrix protein retention in the inter-rod enamel of dental fluorosis [28–31], both of which dramatically affect the wear resistance of the dental fluorosis enamel.

An understanding of the nanomechanical and nanotribological properties of different layers of dental fluorosis is an important contribution of this investigation, as knowledge of such properties can help guide the selection of the appropriate restorative materials to use in clinical practice and promote the development of dental restorative materials. Dental fluorosis enamel presents a distinct gradient nanomechanical behavior that differs from that of the normal tooth enamel. Therefore, the criteria for selecting restorative materials for dental fluorosis enamel are different from those for the normal tooth enamel. Restorative materials with matching nanomechanical and nanotribological properties should be chosen to restore different layers of dental fluorosis enamel.

### Nanomechanical Properties of the Normal and Abnormal Enamel Rods of Dental Fluorosis

**Fig. 7:**
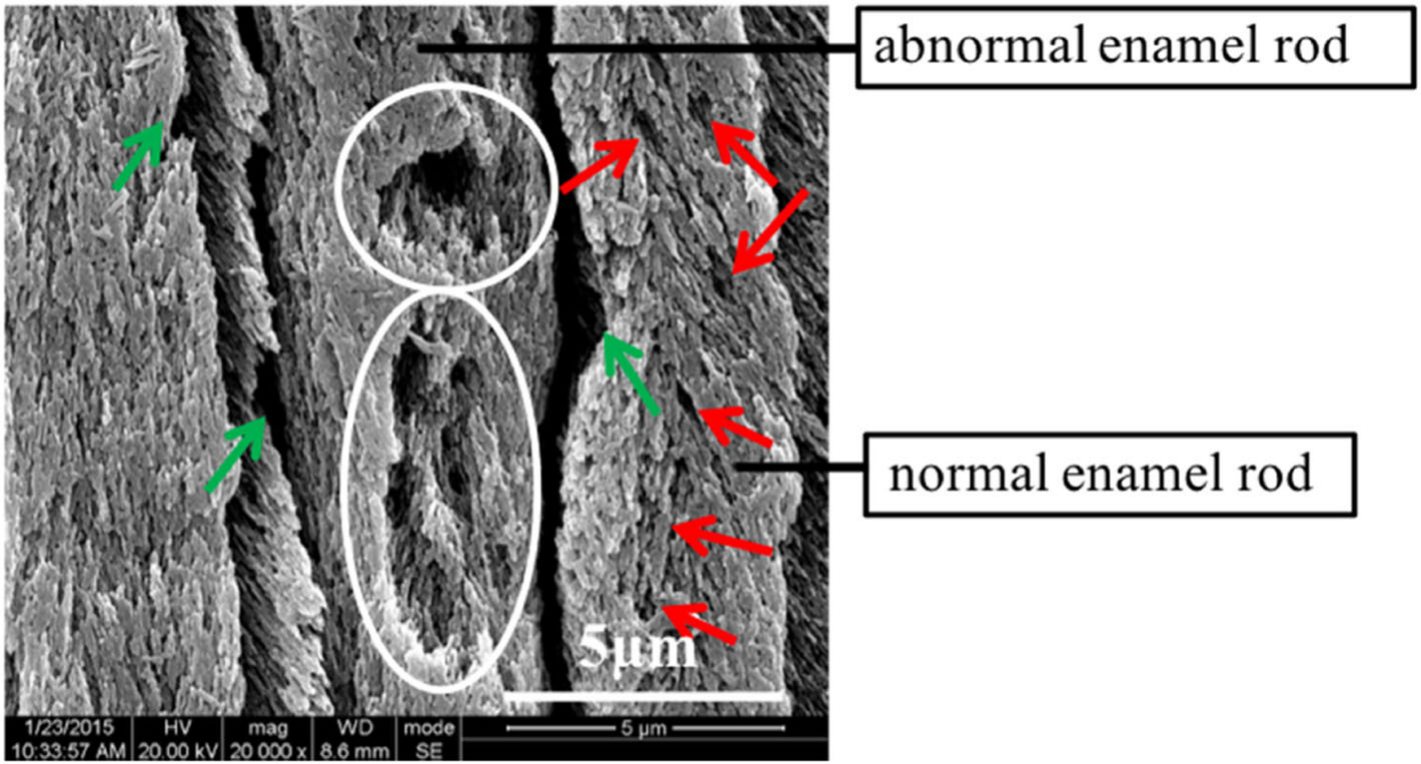
SEM image of normal and abnormal enamel rods in the outer layer of the dental fluorosis enamel. The microstructure is viewed under × 20,000 magnification after etching with 37% phosphoric acid for 30 s. Green arrows show the broadened gaps between the enamel rods, while white circles show the pores. Red arrows indicate loosely arranged crystals in the enamel rods with increasing crystal clearance and micropores

**Fig. 8:**
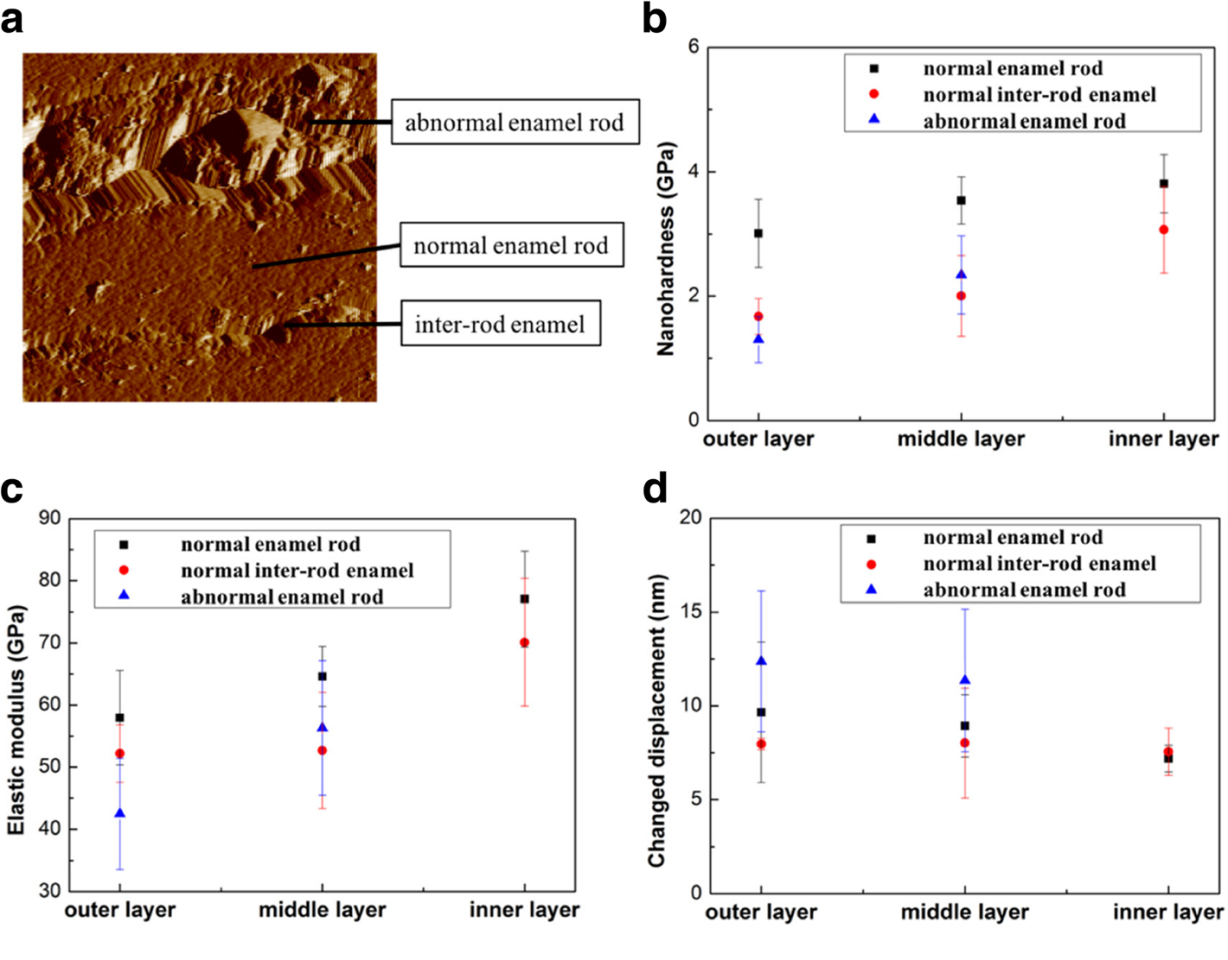
Nanomechanical properties of the normal and abnormal enamel rods of severe dental fluorosis. **a** Normal enamel rod, abnormal enamel rod, and inter-rod enamel are labeled in SPM image of the outer enamel of severe dental fluorosis. **b** Nanohardness. **c** Elastic modulus. **d** Nanoindentation creep behaviors

The nanohardness and elastic modulus of severe dental fluorosis enamel increased from the outer to the inner layers, whereas the changed displacement decreased from the outer to the inner layers. In-depth analysis was subsequently performed to address the large standard deviation of the nanohardness and elastic moduli observed in severe dental fluorosis enamel. The outer and middle enamel layers of severe dental fluorosis can be divided into two types according to the features of their enamel rods, namely, normal and abnormal enamel rods (Fig. 7). Certain enamel rods (i.e., normal enamel rods in severe dental fluorosis) appear complete but exhibit loosely arranged crystal structures and numerous micropores (Fig. 7). Another portion of the enamel rods (i.e., the abnormal enamel rods in severe dental fluorosis) is characterized by numerous pores (white circles in Fig. 7). In this study, the outer and middle layers of severe dental fluorosis enamel presented lower nanohardness and elastic moduli and higher creep deformation than the corresponding layers of the natural tooth enamel, especially in the outer layer. In the outer layer of severe dental fluorosis enamel, normal and abnormal enamel rods showed low nanohardness and elastic moduli and high changed displacement; by contrast, the corresponding features in the abnormal enamel rods were greater (Fig. 8). Studies have suggested that the severity of dental fluorosis is related to the changes in the nanomechanical properties of the teeth [22, 23]. This finding indicates that abnormal enamel rods are seriously affected by excess fluoride element. Given that microstructural changes and poor nanomechanical and nanotribological properties are observed in severe dental fluorosis, restoration is often required to prevent reductions in the vertical distance caused by continuous wear during mastication.

### Appropriate Dental Materials for the Clinical Restoration of Dental Fluorosis

**Fig. 9:**
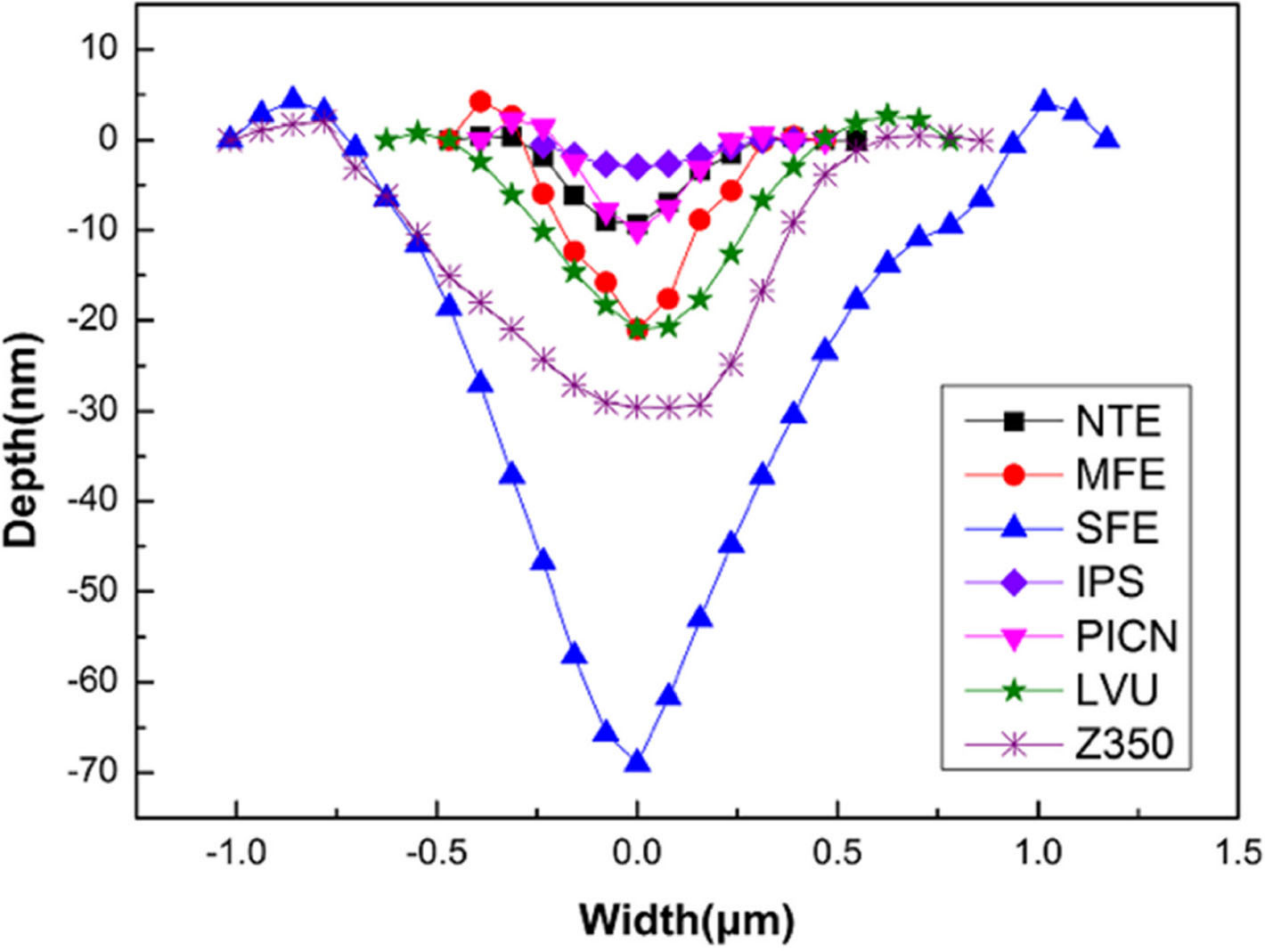
Profiles of nanoscratch tracks on the outer enamel of the normal tooth, mild dental fluorosis, and severe dental fluorosis and four restorative materials. Normal tooth enamel (NTE), mild dental fluorosis enamel (MFE), severe dental fluorosis enamel (SFE), IPS e.max CAD (IPS), polymer-infiltrated-ceramic network (PICN), Lava™ ultimate (LVU), and Fltek™ Z350XT (Z350)

The nanoscratch depths and widths of the outer layers of the normal tooth, mild dental fluorosis, and severe dental fluorosis were compared with those of four restorative materials (Fig. 9). While IPS e.max CAD presented the lowest nanoscratch depth and width, Vita Enamic, polymer-infiltrated-ceramic network (PICN), revealed a nanoscratch depth and width similar to those of the outer layer of the normal tooth enamel. The nanoscratch depth and width of the composite resin block Lava™ ultimate (LUV) were similar to those of the outer layer of the mild dental fluorosis enamel, while the nanoscratch depth and width of the conventional composite resin Fltek™ Z350XT (Z350) were higher than those of the outer layer of the mild dental fluorosis enamel. Among the samples tested, the outer layer of severe dental fluorosis enamel presented the largest nanoscratch depth and width.

Dental fluorosis in the anterior teeth affects the tooth appearance, and severe dental fluorosis with tooth defects in posterior teeth negatively influences mastication [5]. Restorations, such as crowns, inlays, or onlays, are often required to restore the teeth damaged by dental fluorosis [6, 7]. Matching of the mechanical behavior of the restorative material to that of the opposing tooth enamel is especially important to prevent excessive wear of the natural tooth enamel or the applied material itself [8–11]. Ceramics are widely used as restorative materials because of their high biocompatibility and similar esthetics to the natural tooth enamel [47]. However, ceramics present high wear resistance, which causes excessive wear of the opposing natural tooth enamel [47, 48]. Materials with low wear resistance, such as PICN and composite resin block, have been developed as alternatives to ceramics [48, 49]. PICN exhibits a wear resistance similar to that of the outer layer of the normal tooth enamel. Thus, when the opposing tooth is a normal tooth, PICN is the proper material for restoration. However, the opposing tooth in dental fluorosis requiring restoration likely presents mild dental fluorosis. In this case, materials with nanotribological properties similar to those of the mild dental fluorosis enamel are necessary to restore dental fluorosis. Conventional composite resins, such as Z350, reveal a wear resistance lower than that of the outer layer of mild dental fluorosis; such a characteristic may lead to increased wear of the restorative materials. Composite resin block, such as LUV, is fabricated under high temperatures and high pressures and possesses mechanical properties superior to those of the conventional composite resins [50]. In the present study, composite resin block showed a wear resistance similar to that of the outer layer of the mild dental fluorosis enamel. This characteristic implies that this material is appropriate for use as restorative material for dental fluorosis. As the nanomechanical behavior of dental fluorosis enamel determines the selection of the restorative material, the appropriate material should be applied for dental fluorosis to achieve better clinical outcomes. Thus, additional studies on the nanomechanical behavior of dental fluorosis enamel should be conducted, and novel restorative materials should be further developed.

## Conclusion

Based on the results of our analysis, the following conclusions can be drawn:

1. The microstructure and gradient nanomechanical behavior of dental fluorosis enamel were drastically different from those of the normal tooth enamel. The differences were observed in the outer layer of mild dental fluorosis enamel and the outer and middle layers of severe dental fluorosis enamel.
2. Normal and abnormal enamel rods could be observed in dental fluorosis enamel. In particular, the microstructures of abnormal enamel rods in dental fluorosis enamel drastically differed from those of the normal enamel rods. Specifically, abnormal enamel rods displayed lower nanohardness and elastic modulus but higher creep deformation than those of the normal enamel rods.
3. The wear resistance of the composite resin block was similar to that of the outer layer of mild dental fluorosis enamel. Thus, compared with ceramics, composite resin block is a more appropriate restorative material for dental fluorosis.

## Abbreviations

EDJ: Enamel-dentin junction
IPS: IPS e.max CAD
LVU: Lava™ ultimate
MFE: Mild dental fluorosis enamel
NTE: Normal tooth enamel
PICN: Polymer-infiltrated-ceramic network
SEM: Scanning electron microscopy
SFE: Severe dental fluorosis enamel
SPM: Scanning probe microscope
Z350: Fltek™ Z350XT
